# Ageing of Oxygen-Plasma-Treated Polytetrafluoroethylene Surfaces: Revealing a Novel Link Between Morphological Evolution and Wettability

**DOI:** 10.3390/polym18151897

**Published:** 2026-08-02

**Authors:** Rabia Maryam, Ruggero Barni, Hector Eduardo Roman, Claudia Riccardi

**Affiliations:** Department of Physics, University of Milano-Bicocca, Piazza della Scienza 3, 20126 Milan, Italy; r.maryam@campus.unimib.it (R.M.); hector.roman@unimib.it (H.E.R.)

**Keywords:** polytetrafluoroethylene, cold oxigen plasma treatment, wetting, high hydrophobicity, ageing, hydrophilicity

## Abstract

Despite the fact that oxygen plasma treatments are widely used to modify the surface properties of polytetrafluoroethylene (PTFE), the long-term stability of these surface modifications has not been fully investigated. Specifically, the roles of morphological restructuring and chemical modifications at the surface remain to be understood. In this work, we treat commercial PTFE samples using O_2_ plasmas at different discharge pressures to investigate their surface modifications and subsequent ageing at atmospheric pressure. We provide direct evidence that ageing behavior is governed by nanoscale and microscale restructuring of the plasma-modified interface, revealing a novel link between morphology dynamics and wettability properties. To capture this surface evolution, the modified interface was characterized using water contact angle (WCA) measurements, scanning electron microscopy (SEM), Fourier-transform infrared (FTIR) spectroscopy, and mass spectrometry (MS). Initial plasma treatment enhances PTFE hydrophobicity, shifting the WCA from $\theta_{c}≃105{}^{\circ}$ to a highly hydrophobic state of $\theta_{c}≃135{}^{\circ}$. By monitoring the samples in contact with air over a 67-day period a gradual transition toward hydrophilicity was revealed, with WCAs stabilizing at $\theta_{c}≃70{}^{\circ}$ after approximately 20 days. SEM observations identified time-dependent morphological degradation of plasma-induced nanostructures, while qualitative and quantitative FTIR analysis—utilizing the Specified Area Under Band (SAUB) method—confirmed corresponding shifts in carbonyl and hydrocarbon indices. These results demonstrate that ageing kinetics are a direct function of plasma pressure. The transition is further supported by a phenomenological fractal model, which confirms a morphological shift from an initial fractal surface ($d_{s}≃2.25$) toward a standard flat geometry ($d_{s}=2$). Furthermore, calculations indicate a sign reversal in solid-gas interface tension parameters, reflecting the changed chemical nature of the surface. We conclude that the loss of hydrophobicity is driven by a synergistic interplay between morphological relaxation and chemical restructuring.

## 1. Introduction

PTFE is a synthetic fluoropolymer, –(CF_2_–CF_2_)_n_–, produced through the free-radical polymerization of tetrafluoroethylene (TFE). As the simplest perfluorinated alkene, C_2_F_4_ yields a material that is remarkably hydrophobic, characterized by low surface energy, high thermal stability, and low electrical polarizability due to the presence of fluorine atoms [1]. The inherent chemical inertness of PTFE stems from the exceptional strength of the C–F bonds, facilitating critical applications in the biomedical sector [2,3] and the protective coatings industry [4]. Furthermore, various surface treatments can tailor its properties for specialized use in electronics, packaging, aeronautics, and agriculture. While per- and polyfluoroalkyl substances (PFAS) were historically utilized in PTFE production, current manufacturing trends aim to replace these “forever chemicals” with non-toxic alternatives to mitigate their long-term impact on human health and the environment.

*Plasma treatment*: Over the past three decades, extensive research has focused on utilizing plasma techniques to induce surface modifications in polymers, specifically aimed at tuning wetting properties through plasma etching (see e.g., [5,6,7,8]). In particular, while pristine PTFE is inherently hydrophobic, exhibiting a WCA of $\theta_{c}≃105{}^{\circ}$, achieving either highly hydrophobic ($105{}^{\circ}≲\theta_{c}≲150{}^{\circ}$), superhydrophobic ($\theta_{c}≳150{}^{\circ}$) or hydrophilic ($\theta_{c}<90{}^{\circ}$) behavior depends on the specific treatment. Various configurations have been employed to this end, including plasma jets, capacitively coupled plasmas (CCP), ion beams, microwave plasmas, and inductively coupled plasmas (ICP) [9]. These methods are favored for their cost-effectiveness, scalability, and environmental sustainability, as they modify the surface layers without altering the bulk material properties. Plasma treatments transform the surface through a combination of energetic ion bombardment, cross-linking, and localized chemical reactions. Notably, even brief exposure to plasma is sufficient to significantly alter surface wettability [10]. By optimizing discharge parameters—such as radiofrequency (RF) power, treatment time, pressure, and flow rate—and varying the gas composition (e.g., N_2_, CO_2_, Ar, NH_3_, or Ar-H_2_), diverse surface functionalizations can be achieved [11,12,13,14]. Depending on the gas phase chemistry, these treatments can render PTFE either superhydrophobic or hydrophilic, as documented in recent literature [15,16,17].

*Chemical vs. structural modifications*: It is well-established that short-duration exposure to O_2_ or Ar plasma increases the wettability of PTFE without significantly altering its morphology, whereas extended O_2_ treatments induce conspicuous etching [18], however, one expects that both the chemistry and the morphology of the treated surfaces can modify the wettability behavior. Indeed, the authors find that no surface alterations were observed for O_2_ plasmas at short treatment times, and at any treatment times for Ar ones. They conclude that in these cases, surface chemistry is the most important factor in determining the wetting behavior [18]. Research has also indicated that mild radiofrequency Ar plasma treatments, followed by the use of near-UV radiation, lead to substantial surface defluorination and oxidation, thus increasing the hydrophilicity of PTFE without altering significantly the morphological properties of the treated surfaces [19,20,21]. In low-power plasma studies involving O_2_, Ar, N_2_, and NH_3_, all gases were found to induce light etching; however, Ar proved to be the most efficient for inducing chemical modifications, while O_2_ yielded the least significant changes [22]. Interestingly, both dielectric barrier discharges (DBD) and direct or remote Ar plasmas can increase surface energy by introducing polar functional groups, alongside modifying surface structures at the micrometer scale [23,24,25]. Additionally, ion implantation via N_2_ plasma has been shown to reduce the fluorine-to-carbon (F/C) atomic ratio while increasing surface roughness [26,27]. Beyond wetting properties, plasma treatments also offer significant improvements in the tribological performance of PTFE [28,29,30,31]. It has been further established that He and Ar plasmas improve PTFE wettability through either surface defluorination or the incorporation of residual O_2_ [32,33,34,35,36]. Furthermore, at low pressures, PTFE surface sputtering can occur, leading to the deposition of fluorocarbon films onto adjacent substrates [37,38]. Regarding O_2_ plasmas, distinct wetting behaviors have been observed depending on plasma energy [39]. At atmospheric pressure, plasma treatments yield distinct wetting behaviors depending on the specific gas additives used [40,41,42,43], where CO_2_-based plasmas tune PTFE wettability by introducting carboxylic groups enhancing wettability [44].

*Stability and ageing behavior of treated PTFE*: Ageing mechanisms and kinetics in plasma-treated PTFE—stored in either air or phosphate-buffered saline—have been monitored using X-ray photoelectron spectroscopy (XPS), static secondary ion mass spectrometry (SIMS), and dynamic contact angle (DCA) measurements [20]. It is known that O_2_ plasmas can tune PTFE wettability from hydrophilic to superhydrophobic depending on the stabilization of wetting properties [12,15,21,45]. While transitions from hydrophobic to superhydrophobic (and, under harsher conditions, strongly hydrophilic) states have been reported for Ar plasma-treated PTFE [46], recent studies on O_2_ plasma treatment have confirmed the attainment of superhydrophobic states without addressing the long-term stability or post-treatment evolution of these modified surfaces [47]. The ageing of treated PTFE was only partially addressed in [12] since the study focused primarily on how plasma operating parameters, namely RF power and treatment time, influence the initial surface modification. Therefore, the question remains largely open to what extent the post-treatment behavior of the modified PTFE is either stable on long times, or whether it shows an intermediate temporal regime in which the wetting properties change over time (see also [48]). Clearly, the question is relevant to applications and it should be addressed in detail under well controlled conditions.

In this work, we present a systematic study of O_2_ plasma-treated PTFE surfaces, specifically focusing on the post-treatment ageing process under atmospheric conditions, where the evolution of the WCA was monitored over a period of approximately 100 days, considerably extending the results discussed in [12]. In contrast to [12], here the RF power and treatment time were kept constant, while the discharge pressure was systematically varied to isolate its influence on the ageing process. This experimental design shifts the scientific objective from investigating the immediate effects of plasma treatment to understanding the mechanisms governing the temporal evolution of plasma-modified PTFE surfaces during atmospheric ageing. In particular, the present work investigates how the coupled evolution of surface morphology and chemical restructuring controls the progressive changes in wettability and, consequently, the long-term stability of the modified interface.

Utilizing plasma parameters similar to those reported in Ref. [47], we employed a dedicated plasma reactor equipped with mass spectrometry (MS) to characterize the chemical species in the discharge gas-phase prior to treatment. The surface chemical composition and morphology of the samples were tracked throughout the ageing process using Fourier-transform infrared (FTIR) spectroscopy and scanning electron microscopy (SEM), respectively. Our results indicate that all O_2_ treatment pressures initially yield a highly hydrophobic state, with a WCA of approximately 135°. Over time, however, these surfaces undergo a transition toward hydrophilic behavior, eventually stabilizing at a WCA of approximately 70°.

These findings complement existing literature and provide further insight into the mechanisms governing the observed highly hydrophobic-to-hydrophilic transition in plasma-modified PTFE. To further substantiate our findings, we conducted an analytical study of the ageing process using a fractal wetting model recently applied to several polymeric systems [49,50,51]. Our analyses suggest that both morphological relaxation and chemical restructuring drive the observed transition to the hydrophilic regime.

The paper is organized as follows: Section 2 describes the experimental setup and the characterization methods employed. The experimental results are reported in Section 3. In Section 4, a fractal wetting model is applied to describe the observed ageing kinetics of the treated PTFE. Finally, our concluding remarks are summarized in Section 5.

## 2. Experimental Setup and Methods

### 2.1. Plasma Chamber Setup

The O_2_ plasma treatments were performed on PTFE samples (6×6 cm^2^, 1 mm thickness) within a cylindrical vacuum chamber (30 cm diameter, 30 cm height). High-purity O_2_ was introduced into the chamber, which was pre-evacuated using a rotary pump. The discharge was ignited via a RF antenna, coupled through an automated matching network to a 13.56 MHz power supply (Cesar Technologies, Spangenberg-Bergheim, Germany).

The RF voltage was applied to a perforated stainless steel electrode (25 cm diameter), which was electrically insulated from the grounded chamber walls. This configuration generates a diffuse plasma in the volume beneath the electrode, which interacts directly with the samples positioned on the holder at a distance of 65 mm. The gas flows through the electrode’s perforation network in a downward vertical path. Treatments were conducted at three distinct pressures—0.06, 0.1, and 0.2 mbar—with a fixed RF power of 150 W and a treatment duration of 30 min (see Table 1).

**Figure 1:**
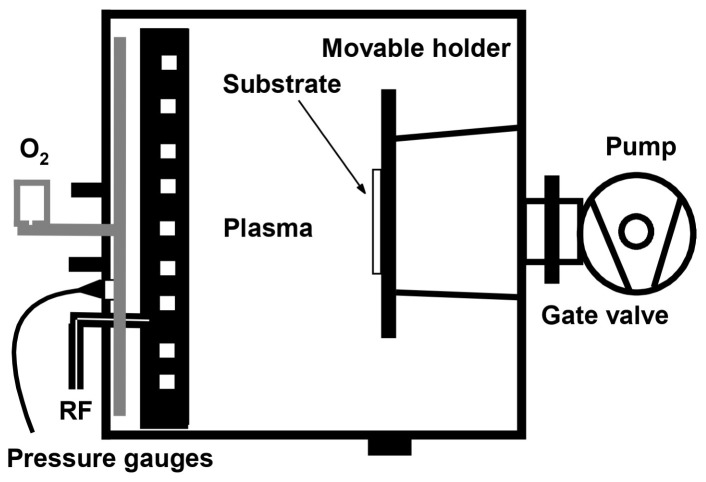
Schematic diagram of the plasma treatment setup. The PTFE samples were positioned on the substrate disc.

Under these conditions, the system operates as a well-mixed reactor, ensuring radial and axial uniformity of the plasma and gas-phase species once the flow exits the thin sheath region adjacent to the electrode. A schematic of the experimental setup is provided in Figure 1.

### 2.2. Sample Preparation and Surface Characterization

The PTFE samples, obtained from commercial PTFE sheets (2.16 g cm^−3^ density—RS-PRO, RS Group plc, London, UK), were prepared by cutting 1 mm thick sheets into 6×6 cm^2^ specimens. Prior to plasma processing, the samples were cleaned with isopropyl alcohol to remove surface contaminants and thoroughly dried. Different techniques are used to characterize the plasma treated samples including contact angle measurements, infrared spectroscopy and scanning electron microscopy [52]. The complete plasma treatments were repeated twice under identical experimental conditions. Similar wettability, SEM, and FTIR results were obtained in both experimental runs, confirming the reproducibility of the treatment.

Surface wetting properties were evaluated before and after plasma treatment by measuring the static WCA using 3 μL droplets of demineralized water. Measurements were performed using an apparatus equipped with SCa20 software (Version 6.1.—DataPhysics Instruments, Filderstadt, Germany). For each sample, the WCA was measured at 6 to 7 distinct locations to determine a representative mean value, ensuring that exactly the same sample region could be monitored at different ageing times. Ageing was induced by exposing the treated samples to air under controlled laboratory conditions.

The chemical evolution of the PTFE surfaces was characterized via Fourier-transform infrared spectroscopy (FTIR-ATR, Nicolet iN10—Thermo Fisher Scientific Inc., Waltham, MA, USA). Spectra were acquired in the (4000–400) cm^−1^ range using an attenuated total reflectance (ATR) mode, with 256 scans performed over 51 s using a cooled detector. To monitor the ageing effect on surface chemistry, measurements were recorded periodically at the selected locations on each sample to ensure data reproducibility and surface uniformity. The surface morphology of both untreated and plasma-treated PTFE was examined using scanning electron microscopy (SEM, Zeiss FEG Gemini 500—Carl Zeiss Microscopy GmbH, Jena, Germany). SEM analysis was conducted at various intervals to track the morphological changes induced by the ageing process over time. For the different ageing stages, SEM micrographs were acquired from the same surface area of each sample used to determine the WCS, allowing to monitor the morphological evolution more accurately.

### 2.3. Mass Spectroscopy

A key methodological feature of this work is the implementation of an *in situ*, operando mass spectrometry framework directly coupled to the plasma reactor. This approach enables real-time monitoring of the chemical species generated during plasma–surface interactions to capture the dynamic mechanisms underlying plasma-induced surface modification. In particular, this configuration allows for the continuous gas-phase tracking of volatile reaction products resulting from oxygen-plasma-driven C–F bond cleavage within the fluorinated polymer chains. Monitoring the temporal evolution of these species provides a direct means to correlate real-time plasma chemistry, etching pathways, and functionalization processes.

A dedicated plasma reactor was utilized for the mass spectroscopic investigation of the discharge gas-phase, featuring a source developed by CCR Technologies GmbH (Troisdorf, Germany) (see [14] for further details). This setup employs an inductively coupled RF discharge to produce a uniform, diffuse plasma capable of operating across a broad range of power levels and pressures.

During analysis, PTFE samples were held at floating potential in the plasma region. Opposite the source, a movable flange connects the chamber to a quadrupole mass spectrometer and ion energy analyzer (EQP-1000—Hiden Analytical Ltd., Warrington, UK) for measuring neutral and charged species (±, up to 1000 amu) and ion energy distributions within ±100 eV. Neutrals were detected using 70 eV electron impact energy (tunable from 4 eV to 150 eV). Species enter through a 100 μm orifice centered on a 50 mm hollow probe cap, where a specialized electrode system ensures efficient collection of neutral fragments and mass/energy-selected ions [14]. The orifice translates up to 300 mm axially for localized probing. Ions traversing the grounded surface’s plasma sheath experience energy shifts determined by the plasma potential [14]; the resulting broad energy distributions dictate average ion energies and high-energy populations driving surface modification.

## 3. Results and Discussion

The experimental results are presented as follows: Section 3.1 details the mass spectrometry of the plasma gas-phase, providing insight into the PTFE etching process and the species generated during the discharge. Section 3.2 reports the surface wettability findings based on WCA measurements. Section 3.3 is dedicated to the SEM analysis of the resulting surface morphologies, while Section 3.4 provides a comprehensive chemical analysis conducted via FTIR spectroscopy.

### 3.1. Mass Spectroscopy of PTFE Etching in O_2_ Plasmas

**Figure 2:**
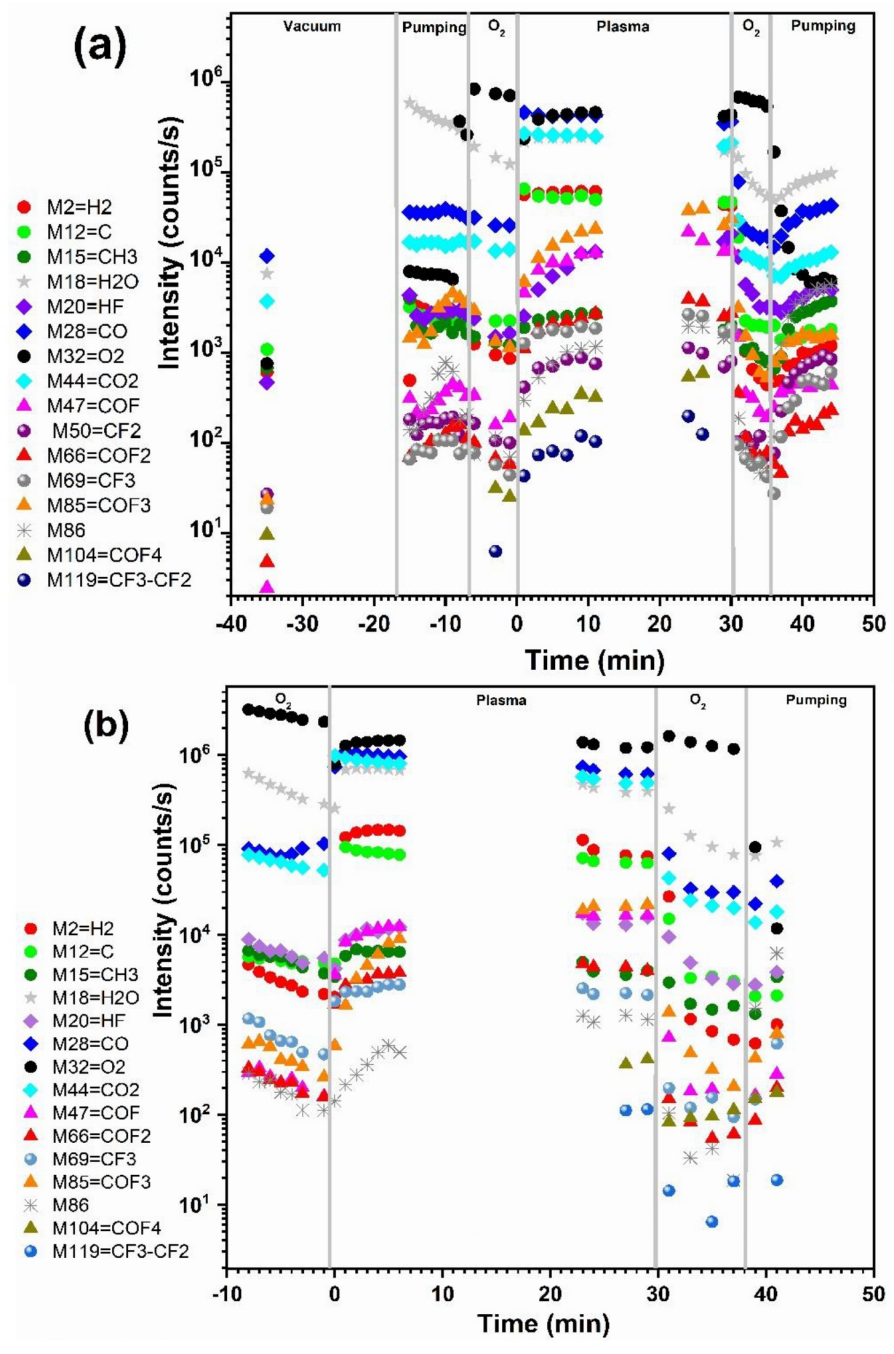
Concentration of main neutral molecules and radicals during the O_2_ plasma etchings of PTFE samples treated at pressures: (**a**) 0.06 mbar (same conditions as Sample 5) and (**b**) 0.10 mbar (same conditions as Sample 1). The vertical lines separate the different regimes employed in the treatment, labelled as: Vacuum (a preliminary measurements of the impurities in the reactor before treatment); Pumping (before the treatment, after the sample was introduced in the device and after the chamber was evacuated before recovering the sample); O_2_ (when the oxygen flow is injected); and Plasma (during the effective treatment in the RF discharge).

Figure 2 illustrates the time evolution of the most significant radicals identified in the gas–phase by the mass spectrometer, when PTFE samples were exposed to O_2_ plasma at 0.06 mbar (Figure 2a) and 0.10 mbar (Figure 2b).

As shown in Figure 2a, prior to treatment, vacuum impurity levels were assessed in the presence of O_2_ flow; aside from minor water vapor outgassing, impurities remained below a few percent. Notably, no fluorine-containing radicals—which would indicate sample degradation or leakage—were detected before discharge ignition. Neutral radical spectra were recorded, and peak areas were integrated to track the evolution of the primary species generated during treatment. To be noted is that O_2_ plasma treatment mainly modifies the outermost surface layer of PTFE by introducing oxygen-containing species and inducing surface etching/reorganization, while the fluorinated PTFE backbone remains unchanged in the bulk (see XPS results discussed in [12]). Thus, wettability variations originate from near-surface chemical and morphological changes rather than complete removal of fluorine.

Upon discharge ignition, a rapid shift in gas-phase composition occurs as the plasma interacts with the PTFE surface. Molecular oxygen levels decline sharply and stabilize for the duration of the 30-min treatment, concurrent with the appearance of primary oxidation products, specifically carbon dioxide (M = 44, with fragments at M = 12 and 28). In contrast, fluorine-containing radicals exhibit a slower evolution, with abundances increasing over the first 15 min before reaching saturation. The most prominent radical detected corresponds to M = 85 amu (COF_3_), likely originating from the oxidation of PTFE chain ends. Conversely, the formation of COF_2_ radicals—which would imply polymer chain scission—appears significantly less probable, particularly as the treatment progresses. The production of COF and, to a lesser extent, COF_4_ radicals was also observed. This suggests that the primary etching mechanism involves the oxidation of specific chain segments.

While oxygen incorporation into the polymer backbone is expected as a corollary to this etching, gas-phase analysis alone cannot confirm surface-bound oxygen. Additionally, a secondary fragmentation pathway was identified at lower concentrations, evidenced by the presence of CF_3_, CF_2_, and C_2_F_5_ radicals. This process is likely mediated by electron impact or physical interactions with ions and neutrals where oxygen is not the direct reactant. Finally, direct fluorine abstraction appears to be a minor contributor, as no significant concentrations of OF, OF_2_, or O_2_F were detected. We also observed indications of discharge-stimulated outgassing and the presence of impurities. Despite the strongly oxidative nature of the gas phase, species such as H_2_, CH_3_, and HF were detected. Furthermore, evidence suggests secondary gas-phase reactions between produced radicals; for instance, the detection of M = 86 amu (tentatively identified as HCOF_3_) likely results from volume reactions rather than direct surface emission from the PTFE.

As shown in Figure 2b, despite that general etching trends remained consistent at higher pressure (0.10 mbar), several distinct differences emerged as compared to Figure 2a. Most notably, the accumulation of COF_3_ radicals was significantly slower and less pronounced, only becoming the dominant etching product toward the end of the treatment. Additionally, the relative abundance of non-oxidized PTFE fragments was higher at 0.10 mbar, despite the increased oxygen concentration. This shift suggests a different balance between competitive polymer fragmentation pathways, likely governed by changes in plasma parameters such as ionization degree and electron temperature ($T_{e}$).

**Figure 3:**
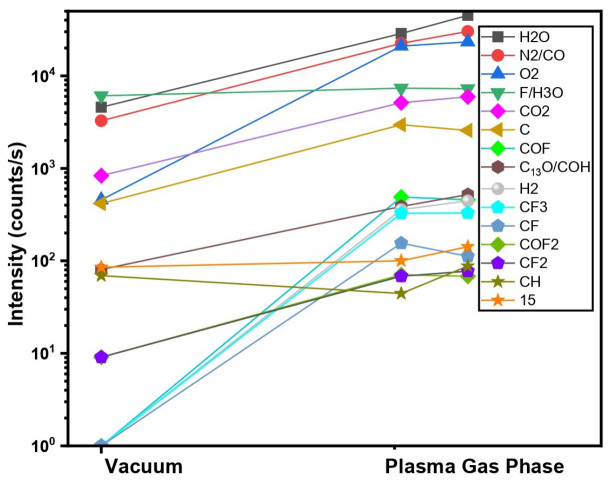
The gas-phase composition measured after 5 min exposure of Sample 5 (cf. Figure 2a).

Finally, a comparison was made with the gas-phase composition sampled directly from the discharge. A small volume of the processed gas was extracted and analyzed upon expansion into the mass spectrometer vacuum chamber (see Figure 3). Although some radical recombination may occur prior to analysis, the resulting spectra are consistent with our observations: a high degree of oxidation evidenced by CO_2_ formation, alongside etching via both oxidized and non-oxidized PTFE fragments. These results strongly support our interpretation of the primary etching mechanisms.

### 3.2. Surface Wettability Analysis Using WCA

**Figure 4:**
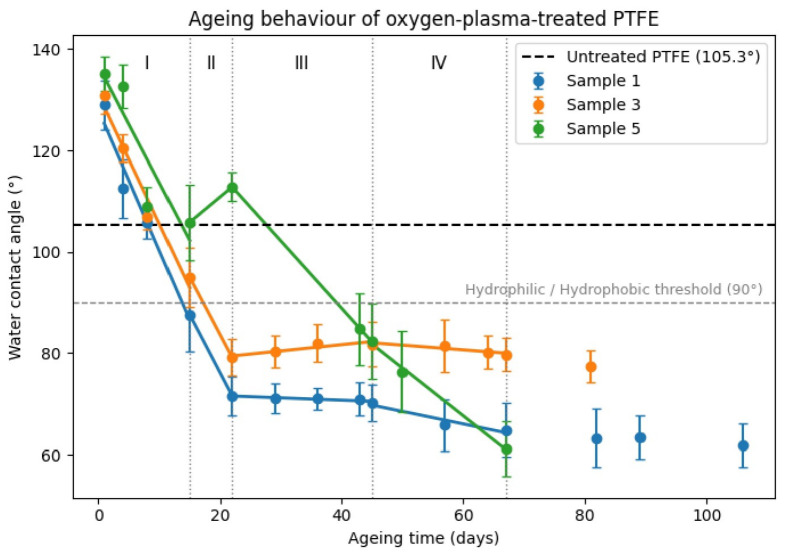
Evolution of the WCA on oxygen plasma–treated PTFE surfaces as a function of ageing time $T_{a}$ [days] for three plasma pressures indicated in the Inset: 0.1 mbar (Sample 1), 0.2 mbar (Sample 3), and 0.06 mbar (Sample 5).

The WCA of the O_2_ plasma-treated PTFE surfaces were measured as a function of ageing time across different discharge pressures (Table 1), as illustrated in Figure 4. For reference, a horizontal dashed line at 105.3° represents the WCA of the untreated PTFE, while a second dashed line at 90° denotes the hydrophobic-hydrophilic threshold. To systematically track the post-treatment surface evolution, the measurements were categorized into four distinct time regimes: Regime I (1–15 days), Regime II (15–22 days), Regime III (22–45 days), and Regime IV (45–67 days). These intervals are demarcated by vertical dotted lines in Figure 4.

In Regime I (1–15 days), all samples exhibited a significant enhancement in surface hydrophobicity immediately following plasma treatment. One day post-treatment, WCAs for all samples remained above 120°, confirming a highly hydrophobic state. This initial behavior aligns with recent findings that oxygen plasma exposure induces superhydrophobicity in PTFE [47]. However, within this initial window, a clear and consistent decrease in WCA was observed across all samples as atmospheric ageing progressed.

Linear fits of the measured WCAs within Regime I yield consistently negative slopes (see Table 2), indicating a rapid transition toward hydrophilic behavior during the initial stage of ageing. Among the treated specimens, Sample 1 exhibits the most pronounced decrease in contact angle compared to Samples 3 and 5. This disparity suggests that the discharge pressure during plasma treatment significantly influences the kinetics of early-stage surface ageing, with higher pressures potentially leading to less stable surface modifications.

In Regime II (15–22 days), the samples diverge in their wetting trajectories. Sample 1 continues its decline, definitively crossing the hydrophilic threshold (90°) with a sustained negative slope. Similarly, Sample 3 exhibits a negative slope, though it remains in closer proximity to the threshold. In striking contrast, Sample 5 displays a positive slope, indicating a partial recovery of surface hydrophobicity during this window—a phenomenon unique to the lowest treatment pressure.

In Regime III (22–45 days), the kinetics for Samples 1 and 3 stabilize, with near-zero slopes indicating a quasi-steady hydrophilic state with minimal WCA fluctuations. For Sample 5, the earlier recovery is followed by a resumption of the hydrophilic trend, with the WCA dropping to 82° at 42 days. To maintain the integrity of our kinetic analysis, the WCA for Sample 5 at exactly 45 days was determined via linear interpolation of adjacent experimental data; this interpolated value was utilized strictly for calculating the slope within this regime.

**Figure 5:**
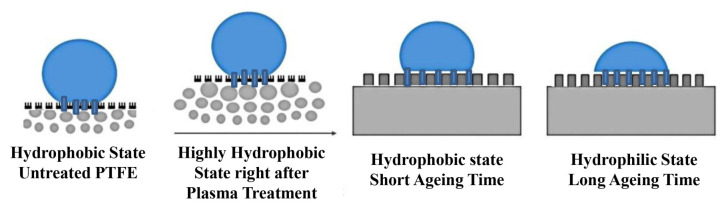
A schematic illustration of the wettability phenomena observed on untreated PFTE, right after O_2_ plasma-treated PTFE, and treated PTFE surfaces in contact with air as a function of ageing time, where a Wenzel type of wetting behavior has been considered.

Finally, Regime IV (45–67 days) captures the long-term stabilization of the plasma-treated surfaces. Samples 1 and 3 exhibit negligible negative slopes, confirming that their hydrophilic behavior has reached equilibrium. However, Sample 5 continues to undergo a prominent decrease in WCA before eventually settling into a hydrophilic state. This persistent evolution suggests that surfaces treated at the lowest plasma pressure remain unsaturated for longer durations, continuing to chemically or morphologically reorganize well after the higher-pressure samples have stabilized. The proposed mechanism for this transition is schematically illustrated in Figure 5.

The observed increase in water contact angle immediately after O_2_ plasma treatment is consistent with previous studies reporting enhanced hydrophobicity of PTFE (see Table 3) due to surface morphology modifications as a result of plasma etching. However, the extent of the increase strongly depends on the plasma conditions, including discharge power, pressure, and treatment time. In Table 3 we also include results using Ar plasma treatments for comparison.

In [12], advancing and receding contact angles were measured, where their difference relates more directly to surface roughness than static WCA alone (whereas for smooth surfaces, they should equal the static WCA). Because of our longer plasma treatment times, we likely incorporated more oxygen into the PTFE surface than reported in [12]. Consequently, the ageing of our treated samples shifted toward an hydrophilic behavior, rather than yielding an increased hydrophobic state as observed in [12].

In addition to the known results shown in Table 3, our study reveals a novel long-term evolution of the WCA which is pressure-dependent and may proceed from an initially highly hydrophobic state to a hydrophilic one under ambient ageing conditions (see Figure 4). The behavior of this pressure-dependent ageing has received limited attention in previous studies and therefore our work provides additional insight into the long-term stability of oxygen plasma-treated PTFE surfaces. Also the role of power needs to be investigated in detail as it can be responsible for some degree of stability in the wetting properties.

### 3.3. Surface Morphology Analysis by SEM

**Figure 6:**
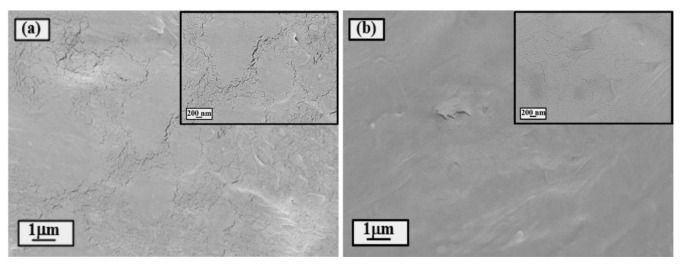
SEM images of untreated PTFE at various ageing times: (**a**) Initial days, and (**b**) after 83 days. The images were obtained at 1 μm scale, and zooms at 200 nm scale are provided in the insets.

SEM was employed to characterize the surface morphology of both untreated and O_2_ plasma-treated PTFE surfaces. Figure 6 displays the SEM micrographs of untreated PTFE recorded at various ageing intervals. To ensure the reliability of our comparative analysis, the untreated control samples were maintained under the same atmospheric ageing conditions as the plasma-treated specimens. These images serve as a baseline to validate the morphological evolution of the treated surfaces over time. As expected, the untreated PTFE displays a predominantly smooth morphology with negligible surface roughness. This lack of significant topographical features is consistent with the intrinsic hydrophobic nature of pristine PTFE, where wettability is primarily dictated by its low surface energy rather than physical structure.

**Figure 7:**
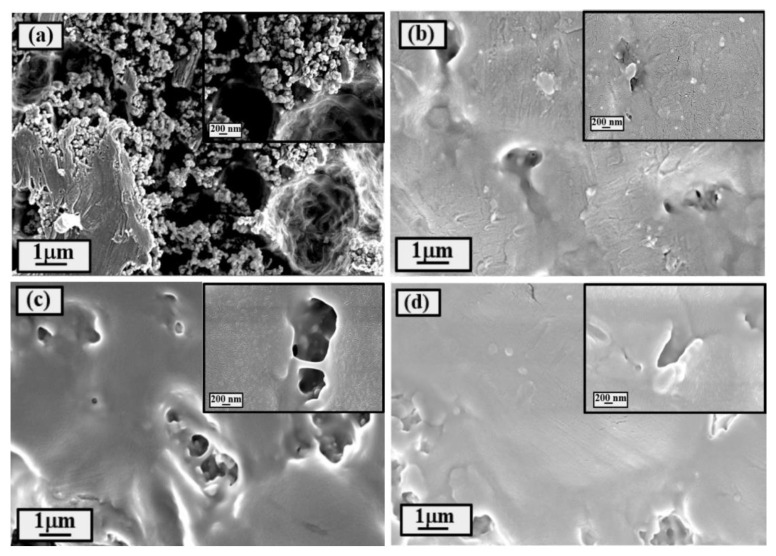
SEM images illustrating the surface morphology evolution of plasma-treated Sample 3 (0.20 mbar) at different ageing stages: (**a**) 3 days (Regime I), (**b**) 21 days (Regime II), (**c**) 39 days (Regime III), and (**d**) 63 days (Regime IV). Images were acquired at 1 μm and 200 nm (Insets) length scales.

Figure 7 illustrates the morphological evolution of Sample 3 (0.20 mbar) across ageing intervals corresponding to the four previously defined regimes. Given that Samples 1 and 3 exhibited similar wetting kinetics, Sample 3 was selected as a representative case to characterize the structural changes. In Regime I (Figure 7a), the surface exhibits prominent hierarchical micro- and nanoscale features. This high degree of roughness can be correlated to the one found for untreated PTFE suggesting that O_2_ plasma etching increases the surface fluctuations by extending the incipient surface fractal dimension of pristine PTFE to larger length scales [50]. In this case, the effective solid-droplet contact area is significantly increased at micrometer scales and a generalized fractal Wenzel model is able to accurately describe the observed dependence of the WCA on surface roughness [49,50,51].

For Regime II (Figure 7b), a noticeable reduction in sharp nano-features is observed, and the surface begins to appear partially smoothed. This gradual restructuring of the surface, driven by interactions with the surrounding atmosphere, reflects the ongoing evolution of the interface toward a new thermodynamic equilibrium. The corresponding morphological relaxation correlates directly with the decline in WCA to approximately $\theta_{c}≃80{}^{\circ}$, marking the transition into the hydrophilic regime.

In Regime III (Figure 7c), the surface continues to homogenize at both the nanometric and micrometric scales. The intricate nanoscale textures present immediately after treatment are largely suppressed. Although the WCA remains relatively stable near $\theta_{c}≃80{}^{\circ}$, the ongoing structural reorganization suggests a process of surface relaxation. Finally, in Regime IV (Figure 7d), the morphology reaches a quasi-steady state, appearing smoother and more stable. The fact that the WCA remains low while the morphology stabilizes suggests that surface chemistry progressively becomes the dominant factor governing wetting behavior in this final phase.

**Figure 8:**
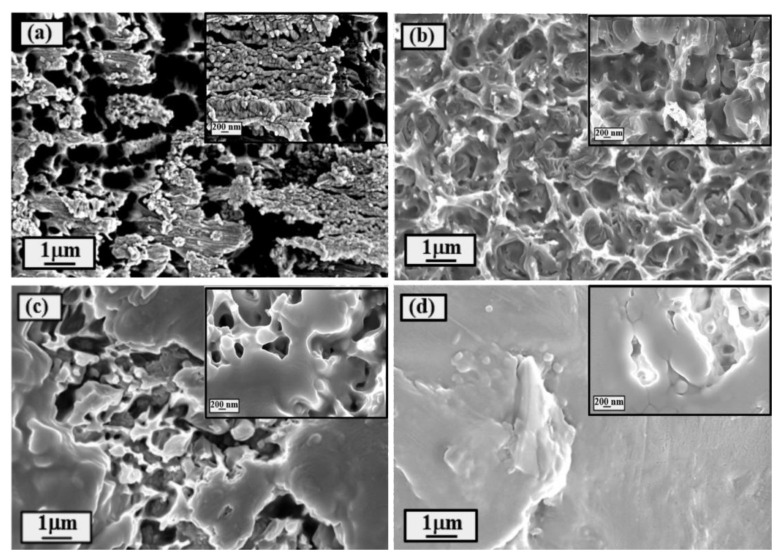
SEM images illustrating the surface morphology evolution of plasma-treated Sample 5 (0.06 mbar) at different ageing stages: (**a**) 3 days (Regime I), (**b**) 22 days (Regime II), (**c**) 30 days (Regime III), and (**d**) 55 days (Regime IV). Images reported at 1 μm and 200 nm (Insets) length scales.

In contrast, Sample 5, treated at the lowest discharge pressure (see Figure 8), demonstrates a distinct ageing trajectory, characterized by a significantly delayed transition from hydrophobic to hydrophilic behavior.

During Regime I (Figure 8a), the PTFE surface exhibits a profoundly altered morphology characterized by significant features at both the nanometric and micrometric scales. In the initial days post-treatment, the surface is populated by a dense distribution of “bumps” and “buds”. This unique topography creates again a hierarchical fractal structure that effectively minimizes the number of contact sites between the water droplet and the solid surface [47,50]. The emergence of these hierarchical features is the primary driver of the initial highly hydrophobic state observed at 0.06 mbar, as they provide the necessary physical framework to support a fractal-type of wetting regime [50], similarly as for Sample 3 (Regime I). This morphology markedly increases the contact angle—reaching $\theta_{c}≃135{}^{\circ}$ in Regime I (Figure 8a)—thereby enhancing surface hydrophobicity toward the observed highly hydrophobic regime.

By Regime II (Figure 8b), the surface begins to show partial relaxation, characterized by the appearance of rounded, continuous, and interconnected structures. However, unlike the higher-pressure samples, the micrometric features persist. This structural durability is sufficient to maintain a strong hydrophobic nature, with WCAs remaining high ($\theta_{c}≃125{}^{\circ}$) even after 22 days. The low-pressure treated PTFE clearly retains these topographical features more effectively than its high-pressure counterparts, resulting in a significantly delayed transition to hydrophilicity.

A visible transition finally occurs in Regime III (Figure 8c). The surface becomes increasingly homogeneous and smooth at the micrometric scale, accompanied by a substantial reduction in nanoscale features. Correspondingly, the WCA shifts to $\theta_{c}≃80{}^{\circ}$, marking the definitive move into a hydrophilic state. At this stage, the wetting behavior ceases to correlate closely with surface topography, suggesting that subsequent surface evolution is primarily driven by chemical reorganization.

In Regime IV (Figure 8d), the surface morphology of Sample 5 undergoes further smoothing and reaches a stable state. With a WCA of approximately $\theta_{c}≃80{}^{\circ}$ after 55 days, the surface demonstrates a stabilized hydrophilic character. A comparable evolutionary pattern is observed across all plasma-treated PTFE samples: the initial surge in WCA is driven by intense morphological modifications at both the micrometric and nanometric scales, followed by a gradual decay in hydrophobicity as these nanostructures dissipate during ageing. However, the low-pressure plasma treatment (Sample 5) delays the morphological evolution prolonging the hydrophobicity for several weeks, after which the long-term hydrophilicity prevails.

After oxygen plasma treatment, the surface of the material continues to change over time at both the nanoscale and microscale. These changes occur because the plasma-activated polymer chains interact with each other and with molecules present in the surrounding air. As a result, the surface structure gradually rearranges, leading to the formation of new nano- and micro-scale features. The observed change in the WCA is a reflection of this slow evolution of the surface, as it continuously adjusts toward a new thermodynamic equilibrium. As the surface moves toward this new equilibrium, its surface energy increases and the initially hydrophobic surface gradually becomes less hydrophobic and more hydrophilic. To be noted, however, is the special case in which the surface energy decreases as observed for Sample 5 within the hydrophobic Regime II shown in Figure 4, suggesting a partial recovery of hydrophobicity. Therefore, the change in wettability over time is directly related to the ongoing structural evolution of the surface. This observation offers a new explanation for plasma-induced wettability changes by showing a direct link between nanoscale surface restructuring and the gradual increase in hydrophilicity.

The observed smoothing of aged samples over time is attributed to the gradual relaxation and restructuring of the plasma-modified surface during atmospheric ageing. This interpretation is supported not only by the SEM observations but also by the FTIR analysis (see Section 3.4), which demonstrates the concurrent evolution of the surface chemistry. Together, these complementary techniques indicate that the ageing process involves coupled morphological and chemical changes rather than simple contamination or imaging artefacts.

### 3.4. Chemical Analysis by FTIR

#### 3.4.1. Qualitative FTIR Analysis

**Figure 9:**
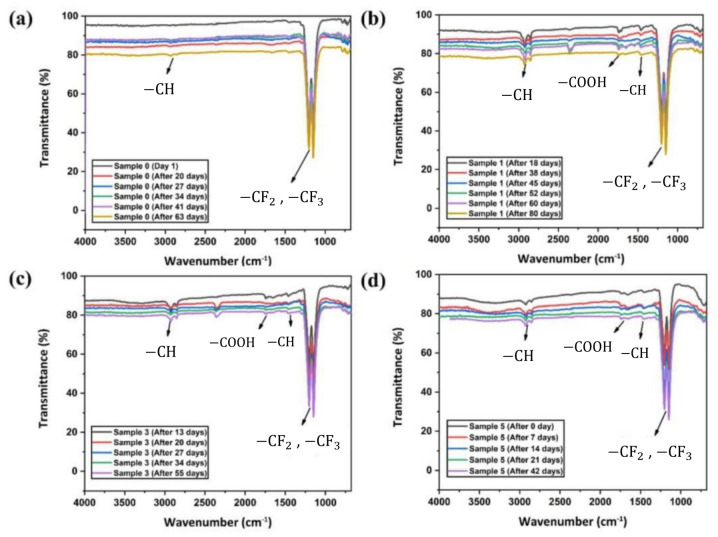
FTIR spectra of PTFE samples, obtained at different ageing times (see insets), showing functional groups at specific wavenumber regions: (**a**) Untreated (Sample 0). (**b**) PTFE treated at 0.1 mbar (Sample 1). (**c**) PTFE treated at 0.2 mbar (Sample 3). (**d**) PTFE treated at 0.06 mbar (Sample 5).

The chemical evolution of the PTFE surfaces was characterized via FTIR spectroscopy in the range of (400–4000) cm^−1^. The resulting spectra for the untreated and plasma-treated samples are presented in Figure 9 at different ageing times.

The spectrum of untreated PTFE (Sample 0), shown in Figure 9a, is dominated by two intense absorption bands at 1149 cm^−1^ and 1203 cm^−1^, corresponding to the asymmetric and symmetric stretching modes of the CF_2_ groups, respectively. Over time, minor bands emerge at 2924 cm^−1^ and 2850 cm^−1^. These features are attributed to C–H stretching vibrations, likely resulting from the adsorption of atmospheric hydrocarbons or moisture onto the polymer surface during ageing.

The spectra of the plasma-treated PTFE samples are shown in Figure 9b–d. A notable absorption band emerged in the (1700–1750) cm^−1^ range, corresponding to the carbonyl (C=O) stretching vibration of oxygen-containing surface functionalities [53]. These oxygen-containing functional groups are incorporated into the polymer backbone through the reaction of residual surface radicals with the oxygen plasma. Additionally, absorption peaks at 1457 cm^−1^ and approximately 2924 cm^−1^ are observed, corresponding to the stretching vibrations of C–H bonds. Notably, these features are absent in the untreated control samples, suggesting that plasma treatment facilitates the subsequent adsorption of ambient species. Consistent across all samples, the dominant peaks at 1149 cm^−1^ and 1203 cm^−1^ represent the asymmetric and symmetric stretching of –CF_2_ (difluoromethylene) and potentially –CF_3_ (trifluoromethylene) groups [54]. Furthermore, the bands observed in the 700 cm^−1^ region are attributed to the wagging modes and backbone chain stretching of the fluorocarbon groups. In addition, a broad absorption band was observed in the 3000–3500 cm^−1^ region, particularly for Sample 5 after ageing. This band is characteristic of O–H stretching vibrations and is attributed to hydroxyl-containing surface species and/or hydrogen-bonded adsorbed moisture commonly observed following oxygen plasma treatment. Because this broad absorption is relatively weak and may include contributions from atmospheric water, it was not considered in the quantitative SAUB analysis. Weak absorption features around 2300–2360 cm^−1^ originate from residual atmospheric CO_2_ within the FTIR optical path. These bands are instrumental artefacts and are unrelated to chemical modifications of the PTFE surface.

**Figure 10:**
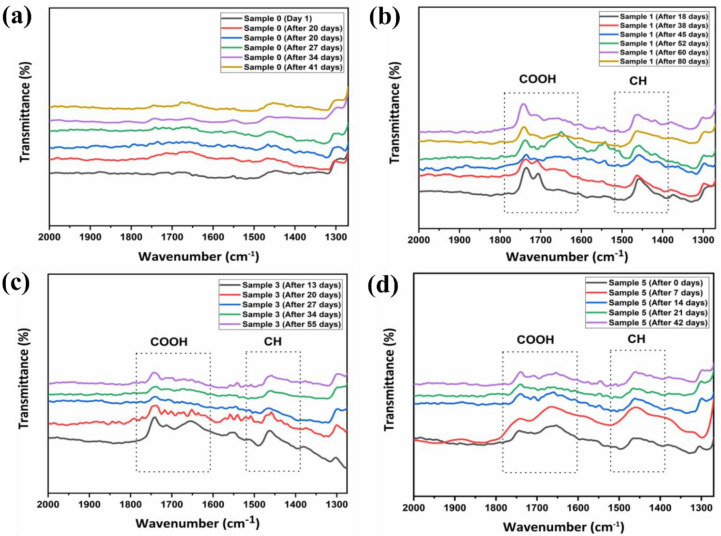
Enlarged view of FTIR spectra (shown in Figure 9) of the region (1300–2000) cm^−1^ indicating the presence of CH and COOH bands in the plasma treated samples as compared to untreated samples: (**a**) Untreated, (**b**) Sample 1, (**c**) Sample 3, and (**d**) Sample 5.

A localized analysis of the C–H and carbonyl (–COOH) regions is presented in Figure 10. For the untreated Sample 0 (Figure 10a), no spectral changes were observed in these specific regions over time, confirming chemical stability in the absence of plasma treatment. In contrast, Sample 1 (Figure 10b) exhibits a pronounced broadening of the –COOH peaks, which correlates with its rapid transition to a hydrophilic state as observed in the WCA measurements. Similarly, Sample 3 (Figure 10c) displays significant –COOH broadening alongside relatively narrow C–H bands, further supporting its hydrophilic character. As previously noted, Sample 5 (0.06 mbar) demonstrates a delayed ageing effect relative to the higher-pressure treatments. This is reflected in Figure 10d, where the C–H bands are notably wider compared to those of Samples 1 and 3. The eventual shift to a hydrophilic state in Sample 5, even as its morphology stabilizes, underscores the pivotal role of long-term surface chemistry reorganization. A detailed quantitative analysis of these chemical shifts is provided next in Section 3.4.2.

#### 3.4.2. Quantitative FTIR Analysis: The Specified Area Under Band (SAUB) Method

Following the qualitative spectral assessment, a quantitative analysis was performed to facilitate a more rigorous comparison of the chemical evolution across the different plasma-treated PTFE surfaces. Since qualitative peak observations alone do not allow for a precise differentiation between samples, we employed the SAUB method to quantify the surface functional groups. Originally proposed for determining the carbonyl index in polyolefins, the SAUB approach integrates the total area beneath a specific functional group’s absorption band rather than relying solely on peak intensity or height [55]. Because the SAUB method is based on integration of the entire absorption band, it is less sensitive to local spectral fluctuations and random noise than peak-height measurements. Prior to integration, all spectra were processed using identical baseline correction and integration procedures to ensure consistent comparison among all samples. This integration method provides a more accurate representation of the total concentration of chemical species, as it accounts for peak broadening—a critical factor in the evolving surface chemistry of plasma-treated polymers.

**Figure 11:**
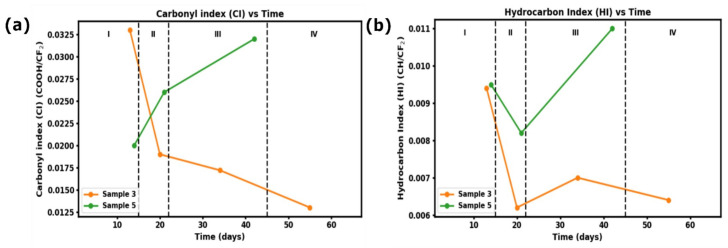
Quantitative analysis using the SAUB method: (**a**) Carbonyl Index (CI) vs. ageing time $T_{a}$. (**b**) Hydrocarbon Index (HI) vs. $T_{a}$.

In this work, the SAUB method was adapted for plasma-treated PTFE by utilizing the fluorinated “fingerprint” region as an internal reference. Specifically, the Carbonyl Index (CI)—defined as the ratio of the area under the carboxyl band to the difluoromethylene band (COOH/CF_2_)—and the Hydrocarbon Index (HI) (CH/CF) were employed to quantify the chemical evolution of the surfaces. Table 4 summarizes the mean and standard deviation (SD) values for both indices across the different treatment pressures. The relatively high standard deviations observed in several samples reflect the inherent heterogeneity of plasma-modified polymers. The oxygen plasma discharge tends to induce non-uniform functionalization and localized surface reorganizations. These significant error margins are consistent with previous literature on plasma-surface interactions, where the stochastic nature of radical-surface collisions often results in varied surface densities of functional groups [56]. A quantitative analysis based on the SAUB method is shown in Figure 11.

In Regime I, both Sample 3 and Sample 5 exhibit non-zero Carbonyl Indices (Figure 11a), confirming that oxygen plasma successfully incorporates polar oxygenated functional groups onto the PTFE surface. Simultaneously, high HI are observed (Figure 11b). Given that untreated PTFE lacks C–H groups, our mass spectrometry data (discussed in Section 3.1) confirms the presence of CH_3_ and other carbonaceous species within the gas phase during the discharge. This provides strong evidence that the C–H signal is a direct byproduct of the plasma treatment rather than post-treatment atmospheric contamination. Despite the presence of these polar carbonyl groups, the WCA remains elevated immediately following treatment. As shown in the SEM analysis, the significant surface roughness facilitates a fractal Wenzel wetting state, which effectively masks the underlying hydrophilic chemistry by preventing the water droplet from fully contacting the functionalized surface.

In Regime II, quantitative FTIR analysis reveals a stark divergence in the chemical evolution of both samples. For Sample 3, both the Carbonyl (CI) and Hydrocarbon (HI) indices decrease relative to Regime I, signaling chemical relaxation. This suggests a partial reorientation or loss of plasma-induced –COOH groups, allowing chemical effects to dominate as initial roughness dissipates and driving a transition to chemistry-dominant wetting. In contrast, Sample 5 exhibits an increased CI while maintaining a high HI, indicating chemical heterogeneity rather than relaxation. Crucially, chemical functionality alone is insufficient to govern Sample 5’s wettability; persistent plasma-induced micro-roughness overpowers the increase in polar groups. This delays the transition to a chemistry-dominated state, leaving Sample 5 in a morphology-dominant regime (Regime II) that causes the observed WCA increase. Conversely, Sample 3 transitions to chemistry-dominant wetting, driven by the relaxation of nonpolar surface contributions and the stabilization of its chemical profile.

In Regime III, the quantitative analysis indicates a definitive transition toward chemistry-dominant behavior, most notably in Sample 5. For this low-pressure sample, both the CI and HI indices reach their peak values during this interval. Rather than representing simple surface relaxation, this pattern suggests an active chemical evolution, potentially through the continued stabilization or late-stage formation of carbonyl functional groups. At this juncture, the high concentration of polar groups effectively outweighs the contributions of non-polar hydrocarbon species, allowing surface chemistry to become the primary determinant of wettability. Consequently, Sample 5 exhibits a sharp decrease in WCA, signaling a clear shift to chemistry-dominated wetting. In contrast, Sample 3 shows a slight decrease in CI and a marginal increase in HI, suggesting that its surface chemistry has largely stabilized with minimal further relaxation. While both samples ultimately enter a chemistry-dominated regime, they do so through different pathways: the high-pressure sample (Sample 3) reaches this state earlier through gradual relaxation, whereas the low-pressure sample (Sample 5) experiences a delayed but intensified chemical activation, resulting in a more abrupt transition toward hydrophilic behavior.

In the final stage, Regime IV, the quantitative analysis indicates that the surface chemistry reaches a quasi-equilibrated state. A slight decrease in CI is observed, likely resulting from the reorientation of polar groups toward the bulk of the polymer or the gradual loss of weakly bound oxidized species. Simultaneously, the HI exhibits a minimal decline, suggesting that the evolution of plasma-induced species has largely subsided. In this regime, surface chemistry remains the dominant factor governing wettability; however, further chemical fluctuations are insignificant, leading to a steady state in wetting behavior. Although FTIR data for Sample 5 was not recorded during this final interval, the indices evaluated at the conclusion of Regime III suggest that chemical stabilization occurs significantly later than in Sample 3. This delayed equilibrium aligns with the prolonged morphological and wetting evolution observed throughout the study for low-pressure plasma treatments.

## 4. Fractal Model for Wetting of Treated and Aged PTFE

### 4.1. Treated PTFE: High Hydrophobicity

Pristine PTFE exhibits an intrinsic water contact angle of $\theta_{c}≃105{}^{\circ}$. Following an O_2_ plasma treatment of duration *T*, the PTFE surface displays enhanced hydrophobic behavior. The evolution of the measured $\theta_{c}$ as a function of treatment time obeys the following empirical relation [50],

(1) $$\cos\theta_{c}\left(T\right)=A\left[1-B\;\frac{R(T)}{1+R(T)}\right],$$

which has be derived from the Young equation [49]. In the above expression, the constants are defined as $A=\gamma_{\mathrm{SG}}/\gamma_{\mathrm{LG}}$ and $B=\gamma_{\mathrm{SL}}/\gamma_{\mathrm{SG}}$, where the parameters γ represent the interfacial tensions between the solid (S), gas (G), and liquid (L) phases. The surface roughness is characterized by the function R(T), which depends on the standard deviation of surface height fluctuations relative to the mean surface level [49,51]. This function is modeled as [50],

$$R\left(T\right)={\left(T/T_{0}\right)}^{\beta },$$

where the exponent $\beta =d_{s}\left(3-d_{s}\right)$ is derived from the surface fractal dimension $d_{s}$, and the parameter $T_{0}$ is a characteristic time constant for the process. In the case of treated PTFE, the evolution of the WCA with *T* is well fitted using the parameter values: A=−0.23, B=−2.4, $d_{s}=2.25$ (yielding β=1.69) and $T_{0}=5.2$ min. Based on this model, after approximately 30 min of plasma treatment, the PTFE reaches a highly hydrophobic state with a WCA of $\theta_{c}≃140{}^{\circ}$ [50]. The present approach corresponds to a ’fractal’ Wenzel-type of model in the case of moderate roughness [49,50,51].

### 4.2. Aged PTFE: Hydrophilicity

**Figure 12:**
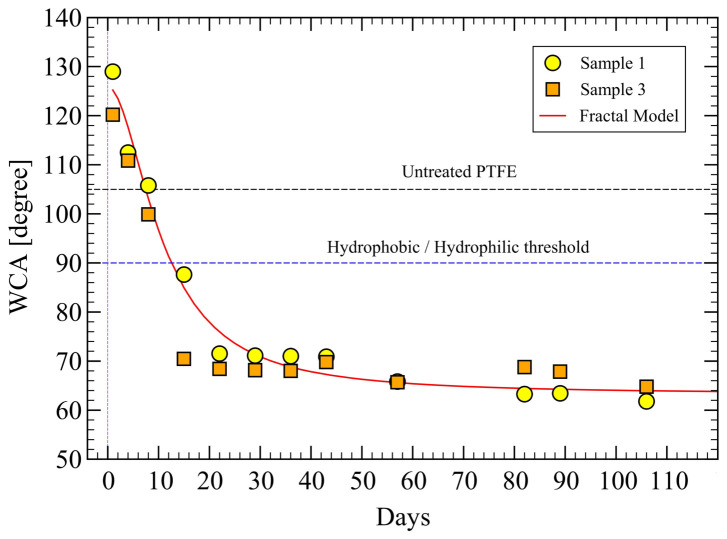
WCA for O_2_ plasma treated PTFE vs. ageing time $T_{a}$ [days]. The fractal model Equation (2) (continuos line) has been applied to Sample 1 (circle) and Sample 3 (square).

After the plasma treatment, the resulting PTFE surface is kept in contact with air and the WCA is monitored periodically over the ageing period $T_{a}$. We observe an evolution of WCA toward smaller values suggesting an hydrophilic behavior, the occurrence of which is expected to be partly due to surface relaxation. The SEM analysis confirms that the aged surface progressively homogenizes, with the fraction of smoothed surface area increasing over time. This evolution of the smooth zones can be quantified by the function $R(T_{a})$. For a perfectly flat surface, the fractal dimension reduces to $d_{s}=2$, yielding a growth exponent of β=2 [50]. Under these conditions, the relaxation function follows the form,

$$R\left(T_{a}\right)={\left(T_{a}/T_{d}\right)}^{2},$$

where $T_{d}$ serves as a characteristic time constant for the decay of surface features. The temporal evolution of the WCA is presented in Figure 12, alongside the experimental measurements for Samples 1 and 3. To model the transition toward a hydrophilic state, we employ the hydrophilic modification of the empirical relation Equation (1) [50],

(2) $$\cos\theta_{c}\left(T_{a}\right)=A\left[1-B\;\frac{1}{1+R(T_{a})}\right].$$

For this regime, the fit yields A=0.45>0 and B=2.3>0. This reversal in sign for both parameters compared to the initial highly hydrophobic state (where A,B<0) signifies a fundamental shift in the interfacial tension energy—specifically at the solid-gas interface given by $\gamma_{\mathrm{SG}}$ (see e.g., Figure 3 in [50])—as the surface undergoes chemical modification. With a characteristic decay time of $T_{d}=11$ days and a growth exponent of β=2, the model accurately describes also the structural modifications with respect to the initial state suggesting the emergence of a stabilized, flat surface (Figure 12).

These results indicate that the newly formed smooth surface regions are predominantly hydrophilic. Consequently, the observed hydrophobic-to-hydrophilic transition in plasma-treated PTFE is a synergistic result of both morphological smoothing and chemical functionalization, both of which are captured within this theoretical framework.

## 5. Conclusions

In this study, we systematically investigated the influence of oxygen plasma processing and subsequent atmospheric ageing on the surface properties of PTFE, with a specific focus on how discharge pressure dictates surface evolution. While all plasma-treated samples initially exhibited highly hydrophobic characteristics ($\theta_{c}≃135{}^{\circ}$), they underwent a consistent transition toward hydrophilic behavior over time ($\theta_{c}≃60{}^{\circ}$–70°). By employing a multi-technique approach—correlating WCA measurements with SEM morphological analysis and quantitative FTIR spectroscopy—we identified a complex, pressure-dependent interplay between physical structure and chemical functionalization.

SEM observations confirmed that oxygen plasma induces significant topographical modifications, resulting in hierarchical micro- and nanoscale structures. These features facilitate a fractal Wenzel wetting state that initially masks the underlying surface chemistry. However, atmospheric ageing triggers a progressive relaxation of these structures. Our theoretical modeling, based on a fractal-to-Euclidean transition ($d_{s}=2.25\to 2.0$), accurately describes this smoothing process. We found that higher plasma pressures lead to faster morphological relaxation, whereas the lowest pressure (0.06 mbar) results in more persistent “buds” and “bumps” that significantly delay the hydrophobic-to-hydrophilic transition. The question whether lower pressures and higher powers may lead to more stable (typically superhydrophic) PTFE remains to be understood.

The chemical evolution, quantified via the SAUB method, revealed the successful incorporation of polar carbonyl (C=O) and hydrocarbon (C–H) groups. Mass spectrometry confirmed that these species are direct byproducts of the plasma-surface interaction rather than post-treatment contamination. The ageing process was characterized by four distinct kinetic regimes, revealing a dynamic competition between surface morphology and chemistry. In the early stages (Regime I), the hierarchical roughness dominates the wetting behavior. As ageing progresses, the dissipation of nanostructures allows the polar functional groups to govern the interface. Interestingly, the low-pressure sample (0.06 mbar) exhibited a delayed but intensified chemical activation in Regime III, reaching a higher Carbonyl Index than its high-pressure counterparts.

Ultimately, both morphology and chemistry reached a quasi-steady state after approximately (45–60) days. These findings demonstrate that plasma pressure is a critical parameter in tuning not just the initial state of a polymer, but its long-term stability and ageing trajectory. By understanding the mechanisms behind this delayed “hydrophobic recovery” and eventual hydrophilic stabilization, this work provides essential guidelines for the design of fluoropolymer surfaces in industrial applications where durable, long-term surface functionality is paramount.

In summary, building upon these insights, this work provides two significant advances in the understanding of plasma–polymer interactions [52]. First, it demonstrates unambiguously a direct correlation between the temporal evolution of surface wettability and the nano- and microscale restructuring of the plasma-modified interface. Indeed, our fractal analysis establishes a direct, quantitative correlation between macroscopic wettability ageing and the underlying physical relaxation of the interface, confirming that gradual morphological rearrangements progressively reduce the hydrophobicity over time. Second, we complement this structural picture by implementing *in situ* operando mass spectrometry directly coupled to the plasma reactor. This setup enabled real-time observation of the underlying chemical reactions, capturing the explicit breaking of C–F bonds and the immediate formation of volatile reaction products in oxygen-containing environments.

Altogether, these theoretical and experimental tracks provide unprecedented insight into the concurrent mechanisms governing plasma-induced surface modification and degradation. Unveiling this explicit link between morphology dynamics and wettability ageing provides a novel framework for understanding polymer interface stability, offering a predictive pathway for tuning the lifetime and performance of plasma-modified materials.

## Figures and Tables

**Table 1:** Sample names and plasma parameters (power, treatment time and pressure).

Sample	Power	Treatment Time	Pressure
Name	[W]	[min]	[mbar]
Sample 1	150	30	0.10
Sample 3	150	30	0.20
Sample 5	150	30	0.06

**Table 2:** Samples (S1, S3, S5) [mbar] and piecewise ageing rates of WCA of plasma-treated PTFE samples. The latter are the mean slope values observed in the four ageing Regimes (I–IV) (cf. Figure 4) in units of [degree/day].

Sample	1–15	15–22	22–45	45–67
Name	[Days]	[Days]	[Days]	[Days]
S1 (0.10 mbar)	−2.77	−2.30	−0.05	−0.25
S3 (0.20 mbar)	−2.55	−2.27	0.12	−0.09
S5 (0.06 mbar)	−2.30	1.00	−1.33	−0.95

**Table 3:** The present results compared with selected ones from the literature on O_2_ and Ar plasma treatments of PTFE. The first column reports the reference, the gas employed in the plasma treatment and the WCA of untreated PTFE. The second column reports the treatment times [min], and the 3rd–4th ones display the plasma parameters, power [W] and pressure [mbar]. The 5th column contains the values of the WCA [°] right after plasma treatments (RAPT), and the last one the WCA measured after ageing in air over a period indicated in days within parenthesis.

Reference	Treatment	Power	Pressure	WCA	WCA
	Time [min]	[W]	[mbar]	(RAPT)	(Ageing Days)
Present work (O_2_) [105°]	30	150	0.06 (S5)	135°	65° (67)
	30	150	0.10 (S1)	128°	79° (67)
	30	150	0.20 (S3)	130°	61° (67)
Kumari (2023) [47] (O_2_) [114°]	30	150	0.05	151°	–
	15	150	0.05	143°	–
	5	150	0.05	132°	–
Zanini (2014) [12] (O_2_) [111°] (Advancing contact angles reported)	10	300	0.09	150°	∼150° (30)
	10	200	0.09	132°	∼150° (30)
	10	100	0.09	125°	∼150° (30)
Salapare III (2013) [15] (O_2_) [119°]	90	400	0.05	151°	–
	60	400	0.05	130°	–
	30	400	0.10	127°	–
Pachchigar (2022) [46] (Ar) [105°]	15	300	0.05	90°	–
	15	200	0.05	125°	–
	15	100	0.05	140°	–
	5	300	0.05	135°	–
	5	200	0.05	135°	–
	5	100	0.05	156°	151° (180)

**Table 4:** Mean values and standard deviations of Carbonyl index (COOH/CF_2_) and Hydrocarbon index (CH/CF_2_) obtained from FTIR peaks measurement for Sample 3 and Sample 5.

Sample	Time	Regime	COOH/CF_2_	CH/CF_2_
	[Days]		Mean (±SD)	Mean (±SD)
Sample 3	13	I	0.033 (0.004)	0.009 (0.001)
Sample 3	20	II	0.019 (0.011)	0.006 (0.001)
Sample 3	34	III	0.017 (0.004)	0.007 (0.002)
Sample 3	55	IV	0.013 (0.004)	0.006 (0.002)
Sample 5	14	I	0.020 (0.008)	0.010 (0.001)
Sample 5	21	II	0.026 (0.006)	0.008 (0.001)
Sample 5	42	III	0.032 (0.015)	0.011 (0.005)

## Data Availability

The data presented here can be obtained upon a reasonable request.
